# Novel Resistance Measurement Method: Analysis of Accuracy and Thermal Dependence with Applications in Fiber Materials

**DOI:** 10.3390/s16122129

**Published:** 2016-12-14

**Authors:** Silvia Casans, Alfredo Rosado-Muñoz, Taras Iakymchuk

**Affiliations:** Department Electronic Engineering, ETSE, Universitat de Valencia, Burjassot 46100, Valencia, Spain; Alfredo.Rosado@uv.es (A.R.-M.); Taras.Yakymchuk@uv.es (T.I.)

**Keywords:** ultra wide range resistance measurement, circuit characterization, moisture content estimation

## Abstract

Material resistance is important since different physicochemical properties can be extracted from it. This work describes a novel resistance measurement method valid for a wide range of resistance values up to 100 GΩ at a low powered, small sized, digitally controlled and wireless communicated device. The analog and digital circuits of the design are described, analysing the main error sources affecting the accuracy. Accuracy and extended uncertainty are obtained for a pattern decade box, showing a maximum of 1% accuracy for temperatures below 30 ${}^{\circ }$C in the range from 1 MΩ to 100 GΩ. Thermal analysis showed stability up to 50 ${}^{\circ }$C for values below 10 GΩ and systematic deviations for higher values. Power supply $V_{i}$ applied to the measurement probes is also analysed, showing no differences in case of the pattern decade box, except for resistance values above 10 GΩ and temperatures above 35 ${}^{\circ }$C. To evaluate the circuit behaviour under fiber materials, an 11-day drying process in timber from four species (Oregon pine-*Pseudotsuga menziesii*, cedar-*Cedrus atlantica*, ash-*Fraxinus excelsior*, chestnut-*Castanea sativa*) was monitored. Results show that the circuit, as expected, provides different resistance values (they need individual conversion curves) for different species and the same ambient conditions. Additionally, it was found that, contrary to the decade box analysis, $V_{i}$ affects the resistance value due to material properties. In summary, the proposed circuit is able to accurately measure material resistance that can be further related to material properties.

## 1. Introduction

Material characteristics can be obtained by means of their impedance. Different measurement instruments and electronic circuits have been developed to obtain the impedance of a certain material or a group of similar materials. For example, the impedance value provides information about liquid conductivity or biological cell suspensions [1], bioimpedance measurement in food processing [2], meat curing processes, meat or fish composition analysis, food freshness determination [3], soil [4], wood [5], sapphire [6], and semiconductors [7]. However, the impedance measurement methods are typically restricted to certain uses and do not provide possibilities of application to different fields.

Contact electrodes are widely used to measure material impedance, and they are inserted into the material so that the material itself becomes part of the electronic measurement circuit, and it can be considered as a sensor whose impedance value can be obtained to provide material information. The accuracy of material impedance measurements depends on the electrode impedances, which appear in series with the material being measured, and on parasitic impedances between the electrodes, the material and its surroundings [8]. Electrode impedance effects can be reduced by using four electrodes, separating potential and current electrodes [9] at an increased circuit complexity.

Accurate impedance measurement is a key factor, and different measurement systems have been proposed: a combination of commercial equipment and specific electronic circuits [10], and a combination of scientific commercial instruments [11,12,13] or specific systems [14,15]. However, the main difficulty in measuring mainly resistive impedance comes when high accuracy and wide range is required, i.e., a single device able to satisfy different uses. Recently, some works have provided different ranges: 0.1 mΩ to 10 Ω [16], 100 kΩ to 100 MΩ [17,18] and 10 kΩ to 1 TΩ [19]. In all cases, they used complex laboratory setups with specific commercial instruments. This is due to the fact that electronic components used in a measurement circuit must be carefully evaluated since they have an ideal behaviour under certain conditions, but the real behaviour can be different, especially in cases where the instruments must work properly for different measurement values, temperature ranges, humidity, etc. Commercial instruments take all these factors into consideration, with the main drawbacks being size, cost, power consumption and portability. Thus, we propose a simple, low power, accurate and wide range measurement to overcome current limitations and adapt to different applications and users.

Considering that improved sensing devices can be developed, this work describes a circuit for resistive impedance measurement in materials—accurate and properly modeled since small variations in the circuit conditions might impact the sensed value. In addition, the electronic circuit must provide flexibility to account for conditions affecting the read value (so that it can be compensated) and convert the resistive impedance value into physical values such as moisture content (widely used in timber), conductivity, etc. [20].

## 2. Objectives

The main aim of the work is to describe and test the accuracy of the resistance measurement in the proposed instrument as a first step to estimate physicochemical properties in materials. Once an accurate resistance value measured is obtained, further variations when measuring materials can be directly associated with material properties and their behaviour.

The proposed measurement device was conceived as a versatile device from which the resistance value can be accurately obtained. Then, using digital techniques, the value can be converted into a physicochemical magnitude from any material or substance. This last step requires specific analysis according to the material and inter-disciplinary work to provide accurate magnitude conversion. In this work, we consider a measurement range between 1 MΩ to 100 GΩ so that a wide range of materials and properties can be covered.

The following goals were proposed:

- Design a new measurement instrument capable of measuring a wide range of resistance values using low voltage supply with improved accuracy compared to other measurement instruments. In addition, it must be versatile to incorporate conversion from resistance value into any material property.
- Develop an automated test bench to allow the characterization of the proposed instrument, and, once characterized, use the instrument in conjunction with the test bench to evaluate material behaviour.
- Electronically verify the instrument under different supply voltages and ambient temperature variations since these parameters must be considered when measuring any material, since they are circuit effects and not directly related to material properties.
- Perform an initial evaluation of the resistance value provided by the instrument under a fiber material. We have chosen timber as a heterogeneous material to obtain the resistance values during a drying process in order to show how the resistance changes as timber is drying.

This paper is organized as follows: Section 3 describes the proposed electronic circuit for resistance sensing, Section 4 shows the test bench and developed software for data acquisition, Section 5 describes the obtained results, Section 6 analyses the results for complex measuring materials (timber), Section 7 analyses the obtained results and Section 8 provides the final conclusions.

## 3. Resistance Measurement: Analog and Digital Circuits

Typically, accurate measurement of high resistance values (above GΩ) must be done using specific instruments as electrometers or megohmmeters/picoammeters, which are not portable due to their size and weight. These instruments use constant-voltage or constant-current measurement methods. In the case of constant-voltage (commonly used), the unknown resistance of the material is connected in series with the electronic circuit and the circulating current is measured, obtaining the resistance value by basic Ohm’s law. However, this is not a simple task since a single electronic circuit cannot guarantee an accurate result for a wide range of resistance values, as it is the case for materials (building materials, textiles, biological tissues, etc.).

With the aim of developing an accurate, wide range, lightweight and small measurement device, we propose an electronic circuit using the latest technology to overcome present limitations. The proposed specifications were the following:

- Ultra wide range resistance measurement: from 1 MΩ to 100 GΩ.
- Resistance measurement is dependent on the temperature. Thus, a temperature sensor must be included to analyse thermal dependence.
- Portability: small size, lightweight and low power.
- Communications: possibility to include different control and visualization options such as local display and keypads (handheld device) or wireless data exchange via different technologies (WiFi, Zigbee, USB). Wireless communication allows creating a remote monitoring network in certain applications where different locations need to be continuously monitored [21,22].
- Data storage: acquired data must be able to be recorded.
- Flexibility: the circuit must be able to be customized for different application areas by software. Thus, the same electronic system can serve for multiple purposes related to resistance measurement (different materials, laboratory use as a characterization/validation bench, remote sensing, handheld device, etc.) by adapting the software.

**Figure 1:**
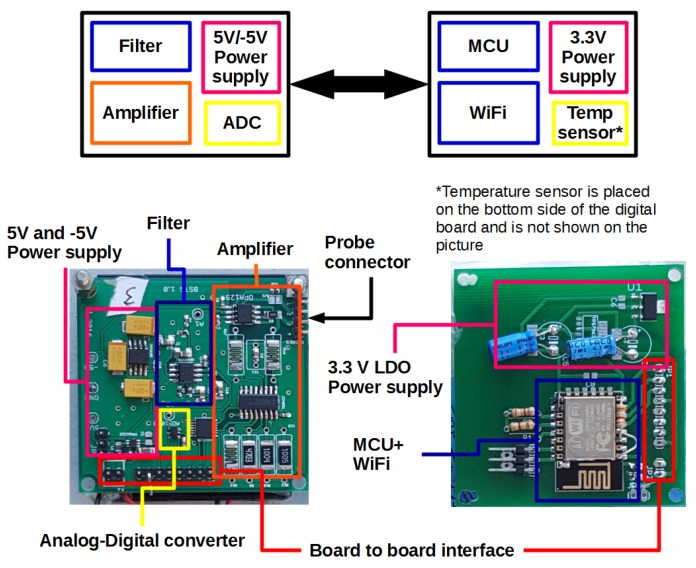
Measurement circuit: external box, internal block diagram of analog and digital parts and analog board showing the internal blocks.

With these requirements in mind, the proposed system depicted in Figure 1 was conceived as an analog section containing the amplification and filtering sections, and a digital part where sensed values from the analog part are read. In addition, the digital part controls the analog section amplification level, the data sampling frequency of analog values, performs digital processing to improve the measured value (averaging, outlier estimation, etc.), converts the resistance value into a physical magnitude (e.g., moisture content in materials), performs the communication with external devices by wireless communication or local display, and stores acquired values.

In order to properly obtain a characterization of the proposed circuit, the following sections describe in detail the analog and digital circuit parts and the test bench used (Section 3.1, Section 3.2 and Section 4), with the main focus on the analog part, responsible for accurate and wide range measurements.

### 3.1. Analog Circuit

When values of the resistance to be measured are above 1 GΩ, different effects on the circuit cause a variation in the obtained value from the real value. The main undesired effects are caused by offset voltage, polarization currents in the operational amplifiers, leakage currents, noise, etc. Thus, it becomes very important to obtain a characterization of the circuit in order to account for these effects and compensate them according to the measurement conditions (resistance value and temperature) and the circuit itself.

**Figure 2:**
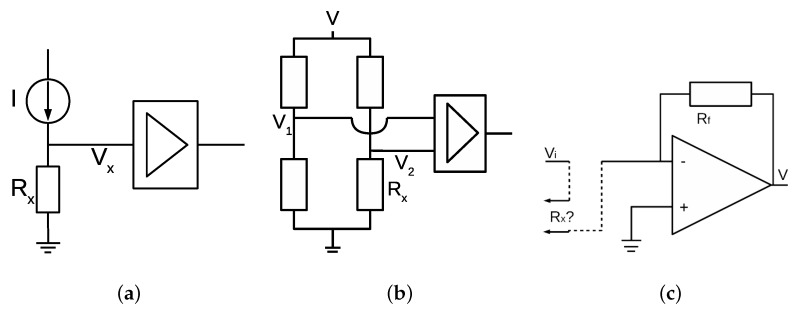
Three possibilities for resistance measurement. (**a**) current source; (**b**) wheatstone bridge; (**c**) inverter circuit with ideal amplification stage. This was the selected measurement circuit, and $R_{x}$ is the unknown resistance value from the material to be measured.

Different circuit configurations might be used in electrical resistance measurement. The proper configuration depends on the resistance range to be measured. Figure 2 shows three possibilities considered. The circuit in Figure 2a uses a current source and voltage $V_{x}$ is amplified and measured. In this case, as the requirements are low voltage and wide resistance range for $R_{x}$, it is common that the current source is not stable for precise pA generation and the amplification section must be programmable to adapt different ranges, increasing complexity without precision guarantee. Figure 2b shows a second option considering a wheatstone bridge, $R_{x}$ being one of the resistors in the bridge. Differential voltage $\left(V_{2}-V_{1}\right)$ is measured and amplified by a varial gain stage. An instrumentation amplifier with fA polarization current is required to avoid load effects. Thus, a discrete instrumentation amplifier needs to be designed by using electrometer-grade discrete operational amplifiers, which increases complexity. Finally, the selected option was an inverter circuit (Figure 2c), as it is simple, low-cost and effective, with the single requirement of using an electrometer-grade operational amplifier to avoid load effects due to polarization currents. The amplification section of the circuit shows a fixed voltage $V_{i}$ applied and $R_{x}$ as the unknown resistance value. Equation (1) provides the formula relating all values involved in the measurement circuit: input voltage $V_{i}$, output voltage $V_{o}$, feedback resistor $R_{f}$ and measured resistance $R_{x}$. For a certain value of $V_{o}$, $R_{x}$ can be obtained if $V_{i}$ and $R_{f}$ are known, as is the case:

(1) $$V_{o}=-\frac{R_{f}}{R_{x}}V_{i}.$$

To avoid operational amplifier saturation and obtain the maximum output voltage ($V_{o}$), the proposed circuit includes a multiplexer in the feedback loop of the operational amplifier to modify the gain by changing the $R_{f}$ value according to $R_{x}$. Three different $R_{f}$ values are included, and the multiplexer is controlled by the digital part . For the $R_{x}$ range [1,50] MΩ, $R_{f}=680\;K\Omega$; for $R_{x}=\left[50\;M\Omega ,3\;G\Omega \right]$, $R_{f}=33\;M\Omega$, and, for $R_{x}=\left[1.5,100\right]\;G\Omega$, $R_{f}=1\;G\Omega$. An automatic undervoltage detection procedure is followed to automatically select $R_{f}$, where voltage from the amplifier is in the [0.6,3.5] V range.

In this case, the multiplexer must be carefully chosen since its real behaviour can be very different from the ideal expected working mode [23], as described later. After the amplification section, a low pass filter (Sallen–Key) to remove noise contributions is included.

However, this ideal behaviour does not occur in the real circuit. Real behaviour is ignored as the undesired effects are negligible for low $R_{x}$ resistance or high $V_{i}$ voltage values. Actually, a measurement technique for some instruments consists of increasing $V_{i}$ (up to 1000 V in some cases) when $R_{x}$ increases [24] so that undesired effects are always negligible. In the proposed circuit, due to the design consideration of low power, voltage $V_{i}$ is fixed (5 V) for all resistance measurements, and thus real behaviour must be considered since it is not negligible for high resistance. This causes, for a resistance value of $R_{x}=100\;G\Omega$, that the circulating current is on the order of pA. Thus, the proposed circuit must be carefully characterized since the following factors must be considered:

- Real behaviour of electronic components used in the circuit.
- Effects of the printed circuit board (PCB).
- Leakage currents. Leakage currents are generated by stray resistance paths between the measurement circuit and nearby voltage sources. These currents can degrade the accuracy of low current measurements considerably. Guarding is used as an effective way to reduce leakage current [25], but leakage still exists.
- Electrostatic effects.
- Noise sources associated to electronic components. Its contribution is increasing with temperature, high resistance values and amplification level. This can be a limiting factor in measurement, as noise can hide measured value from $R_{x}$ [26]. On average, this noise effect is zero, but instant values can cause saturation in the amplifier.

**Figure 3:**
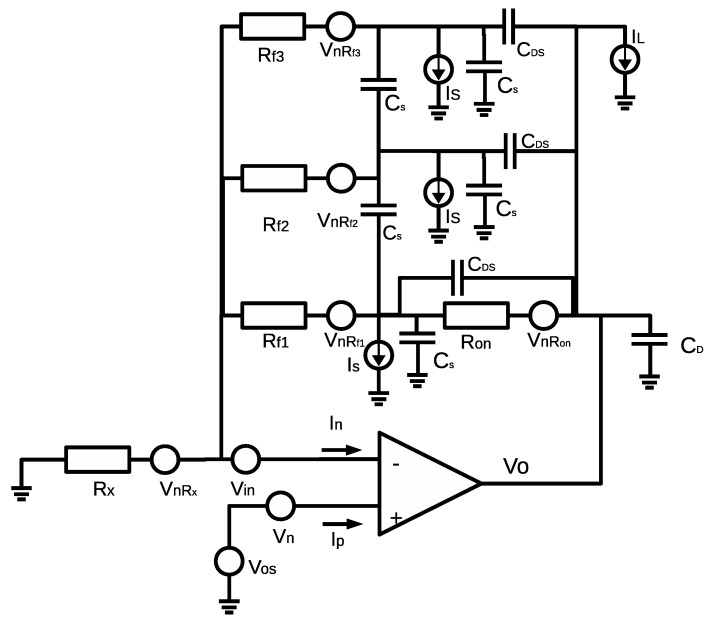
Model of the input amplifier section considering real effects of electronic components used.

The simplified real model for the input amplifier is shown in Figure 3 for three different feedback resistor values ($R_{f1},R_{f2},R_{f3}$) considering both AC and DC models of the multiplexer, $C_{D}$ being the output pin capacity of the multiplexer, $C_{S}$ being the associated capacity for the input pin in each internal switch of the multiplexer, $C_{DS}$ being the capacity between multiplexer pins, $C_{SS}$ being the parasitic capacitance between multiplexer channels, $I_{S}$ being the leakage currents in the multiplexer pins, and $I_{L}$ being the leakage current at the multiplexer output terminal. With all of these real effects, Equation (1) is not adequate, especially for certain values of $R_{x}$ and $R_{f}$ due to:

- Thermal noise in the resistors of $V_{nRf_{i}}$ increasing with temperature and being more important as resistor values increase [25].
- Operational amplifier noise $V_{n}$ due to the offset voltage between input pins $V_{os}$, and noise $V_{in}$ due to the polarization currents $I_{p},I_{n}$ [26].
- Leakage currents in the multiplexer $I_{L},\;I_{S}$, parasitic capacitances $C_{DS},\;C_{D},\;C_{S}$ and in resistance of the multiplexer $R_{on}$ [23].

As seen in Figure 2 and Figure 3, the real model differs from the ideal circuit. Thus, the circuit response must be evaluated according to the real circuit, especially in cases where the current error sources are comparable in magnitude to the currents to be measured (in the case of GΩ measure values, the current to be measured is on the order of pA). In order to make a proper selection in the electronic components being part of the analog circuit so that the accuracy can be guaranteed, we need to evaluate possible error sources. Using the superposition theorem, we can separate the AC and DC models in the circuit:

#### 3.1.1. DC Analysis

In the DC model, the error sources come from $V_{os}$, $I_{p}$, $I_{n}$, $I_{S}$ and $I_{L}$. The parasitic capacitance can be considered as open circuits. Then, the circuit in Figure 3 is simplified. Additionally, using an electrometer-grade operational amplifier, $I_{n}$ contribution can be ignored since it is much lower than $I_{S}$ and $I_{L}$. Thus, we have the error sources due to $V_{os}$ (Equation (2)), $I_{os}$ and $I_{b}$ (Equation (3)):

(2) $$V_{o}=\left(1+\frac{R_{f}+R_{on}}{R_{x}}\right)V_{os}+\left(I_{b}-\frac{I_{os}}{2}\right)\left(R_{f}+R_{on}\right),$$

(3) $$I_{os}=I_{p}-I_{n}\;;\;I_{b}=\frac{I_{p}+I_{n}}{2}.$$

Considering that the resistance measurement range is [1 MΩ...100 GΩ] and $R_{f}\gg R_{on}$, we can deduce that $V_{o}$ contribution due to DC error sources depends on the $R_{f}/R_{x}$ ratio. Thus, if a wide range of measurement values is required (as in this case), different $R_{f}$ resistors must be used in the circuit so that the ratio is always low and the error sources can be neglected. However, the ration cannot be arbitrarily small since it is the amplification/attenuation ratio for $V_{o}$ (Equation (1)). Moreover, if we intend the circuit to be valid for a wide temperature range, we must evaluate the thermal dependence. Voltage $V_{o}$ is affected by $V_{os}$ and $I_{b}$ because both depend on temperature (Equations (4) and (5)):

(4) $$V_{os}\left(T\right)=V_{os}\left(25{}^{\circ }C\right)+TC\left(V_{os}\right)\left(T-25{}^{\circ }C\right),$$

(5) $$I_{b}\left(T\right)=I_{b}\left(25{}^{\circ }C\right)2^{\left(\frac{T-25{}^{\circ }C}{10}\right)},$$

where $TC(V_{os})$ is the thermal coefficient for the offset voltage and $I_{b}\left(25{}^{\circ }C\right)$ is the polarization current at 25 ${}^{\circ }$C.

#### 3.1.2. AC Analysis

In general, the noise in the circuit is not an issue as its mean value is null. However, it might be a cause of saturation in $V_{o}$ as the instant value of noise is superimposed to the acquired signal. In this case, we must consider the error sources due to electrical noise associated with the operational amplifier and thermal noise associated with resistors (Equations (6)–(8)). These equations show the density of noise voltage at the operational amplifier input $\sigma_{n}$ (6), and the density of the noise current at the input $\gamma_{n}$ (7). The noise voltage density in a resistor due to temperature $V_{nR}$ is given by Equation (8):

(6) $$V_{n}=\sqrt{\sigma_{n}^{2}B},$$

(7) $$i_{n}=\sqrt{\gamma_{n}^{2}B},$$

(8) $$V_{nR}=\sqrt{4KTRB}.$$

*K* being the Boltzmann constant ($1.38\times 10^{-23}$ J/K), *T* being the temperature in Kelvin degrees, and *B* being the bandwidth.

From previous equations, we observe that the noise in the circuit will increase with the resistor values and temperature. Consequently, the maximum $V_{o}$ value must be reduced if noise is high, in order to avoid operational amplifier saturation. Otherwise, in the case of ultra high $R_{x}$ values, the output value will not be accurate due to the high presence of noise.

Despite these effects needing to always be considered, it forces the design to use an electrometer-grade operational amplifier to reduce undesired errors and make a proper selection for all the electronic components to reduce error contribution in the amplifier output voltage $V_{o}$. Table 1 shows the chosen characteristics (at 25 ${}^{\circ }C$) of resistors, the multiplexer and the operational amplifier.

However, the input amplifier can be corrupted by noise and the output voltage $V_{o}$ serving as input to the filter section can be saturated. As supply voltage is 3.3 V from the digital board, ±5 V power supply is incorporated in the analog board. Thus, the maximum theoretical output voltage is ±3.5 V due to the saturation voltage in the operational amplifier. Then, for $R_{x}$ values above 10 GΩ, the maximum output voltage is greatly reduced since the saturation voltage is very low due to noise contributions, and the gain value must be reduced to avoid signal saturation. For this reason, the filter circuit after the amplification section allows obtaining an adequate output voltage after filtering.

### 3.2. Digital Circuit

To minimize interferences, the analog and digital parts were separated into two boards in addition to making the design more flexible since the digital board can be designed for different needs while maintaining the analog board. Currently, two digital boards are tested for two applications: a wireless sensing node and a handheld measurement device. The interface between digital and analog boards consists of eight pins: two ground pins, three analog multiplexer control lines for gain control, $I^{2}C$ bus interface for the A/D converter and one analog power shutdown line to switch on and off analog power supply in order to minimise power consumption. All lines of the interface are 3.3 V. The main component of the digital board is the ATmega 328 8-bit microcontroller (Atmel Corp., San Jose, CA, USA), powered through the AMS1117 low-drop voltage regulator (Texas Instruments Inc., Dallas, TX, USA) to provide stable 3.3 V to its components.

The wireless sensing node board used in this work includes an ESP12E Wi-Fi module (Espressif, Shanghai, China). This module is a complete wireless solution and communication stack for communication through IEEE 802.11 b/g/n networks. The module can work in Access Point (AP) or client modes. In the client mode, it connects to the established network and sends cyclic reports on a remote server. Using such a configuration, a wireless sensor network with multiple nodes can be quickly developed over an existing Wi-Fi infrastructure. In the AP mode, the node creates its own Wi-Fi network and allows users to connect from any Wi-Fi enabled device. After the connection, the user can access a simple web interface in a browser, providing real-time data. The handheld version of the digital board contains a USB CDC (Communication Device Class) interface, an HD44780 20×4 display LCD (Hitachi, Tokyo, Japan) and four buttons as a user interface. It can register up to 1024 measurements and store them in the non-volatile memory of the ATmega 328 microcontroller, which can be downloaded to a PC through the USB connection using the virtual serial port configuration.

For each resistance value result displayed or recorded, the microcontroller calculates the instant resistance value from the last 10 measurements (averaged over a sliding window). Then, using pre-calculated property-resistance curves together with the proper temperature correction, a physicochemical value of the material can be obtained. The type of the material (and thus the corresponding conversion and correction curves) can be selected by a user through the screen menu in the handheld device or through the wireless server configuration interface.

## 4. Characterization Test Bench

In order to verify the functionality and characterize the circuit, a reduced size test bench was developed with two main aims: flexible and configurable user interaction, and adaptation to the tested device. The test bench consists of the measurement circuit and a digital connection with a computer (WiFi, Zigbee or serial wired) where a LabVIEW (2015 version, National Instruments, Austin, TX, USA) interface was done. The graphical interface provides monitoring and data recording to allow:

- Accuracy analysis in the measurement range.
- Evaluate the most adequate supply voltage $V_{i}$ for the analog circuit part of the device.
- In combination with a climate chamber, obtain characterization data of the device with variable temperature and ambient humidity.
- Report generation and data analysis.

**Figure 4:**
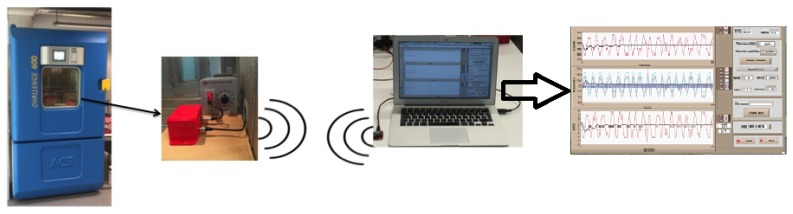
Test bench used for the circuit characterization. The circuit is connected to a PC for monitoring and data analysis using a digital communication system (wireless or wired), and its behaviour for different temperatures and ambient humidity is analyzed by introducing it in a climate chamber.

Figure 4 depicts the test bench structure where the device is connected to the PC for data exchange, and, inside the climate chamber, the circuit characterization is carried out. In this case, the circuit is characterized and calibrated using a resistance decade box as the pattern, but the same test bench can also be used to characterize the circuit behaviour with materials (wood, sand, textiles, etc.). The user interface also shown in the figure allows controlling the gain level in the circuit, monitoring temperature and ambient humidity with an accuracy of $\pm 0.5{}^{\circ }C$ and ±1% RH and monitoring the temporal evolution of the circuit output voltage $V_{o}$ with its equivalent resistance value ($R_{x}$) and the relative error in the measured value $\epsilon_{r}$ (if a pattern resistance is used). It also displays the associated extended uncertainty $\sigma_{ext}$ and accounts for thermal coefficients of pattern resistors to allow accurate thermal analysis of the circuit.

## 5. Experimental Results

The proposed circuit is analyzed and characterized under real conditions of varying sensing values of $R_{x}$, temperature and humidity. These results allow obtaining a deep knowledge of the circuit, its limitations and possible compensations to be done. Evaluation of accuracy, extended uncertainty of $R_{x}$ measured values and thermal characterization was done.

### 5.1. Evaluation of Accuracy and Extended Uncertainty of $R_{x}$ Measured Values

To evaluate long-term accuracy, Type A extended uncertainty was obtained [27], where n=32 independent observations (measurements) were obtained from a calibrated decade box, two per day (every 12 h) for 16 days in our laboratory. During the 16-day period, the temperature and relative ambient humidity range was $\left[23,\;25\right]{}^{\circ }C$ and [43, 55]%, respectively. Each of the observations was obtained by averaging 100 readings for two minutes. Table 2 shows the results, showing the nominal value of the measured resistance ($R_{nom}$), the calibrated resistance value ($R_{cal}$) and its tolerance, the obtained value using the circuit ($R_{m}$), the extended uncertainty $\sigma_{ext}$ and the relative error $\epsilon_{r}$ when comparing the result with the calibrated value $R_{cal}$. As seen in the table, accurate results are obtained even for 100 GΩ, where 0.7% relative error and ±1 GΩ extended accuracy was obtained, especially when considering that low voltage $V_{i}=5$ V is used.

These results guarantee that the device can measure resistance with a relative error lower than 1%.

### 5.2. Thermal Characterization of the Measurement Circuit

Temperature variations affect the resistance of a certain material but also the circuit response due to the effects of electronic components. This analysis was done to evaluate the effect of temperature in the electronic components of the circuit since high resistance measurement deals with very low values and small temperature variations need to be taken into consideration. In order to fix ambient conditions, a climate chamber was used and temperature analysis was done at $10{}^{\circ }C,\;20{}^{\circ }C,\;30{}^{\circ }C,\;40{}^{\circ }C$ and $50{}^{\circ }C$ with a constant value of 50% ambient relative humidity. Once the thermal equilibrium is achieved by the climate chamber, data were acquired every three seconds, averaging them for five minutes (100 samples). In total, for each temperature and $R_{cal}$ value, measurements were recorded for 30 minutes. The measurement circuit and calibrated resistance decade box were introduced in the climate chamber and measured values sent to a computer via wireless connection (Figure 4). Table 3 shows the obtained results for accuracy and extended uncertainty in the range of resistance values [1 MΩ...10 GΩ]. As seen, a maximum error of 0.4% is obtained for 10 GΩ resistance value.

**Figure 5:**
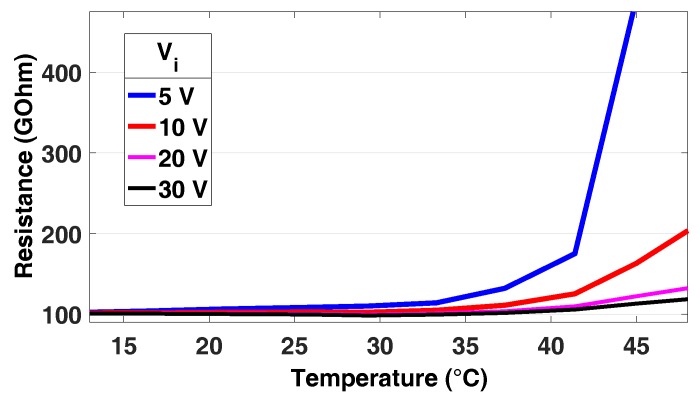
Measured value for $R_{x}=100\;G\Omega$, for $V_{i}$=5 V, 10 V, 20 V and 30 V. Accuracy decreases for low $V_{i}$ values when resistance is very high.

As seen in Table 3, the error in the measurement is lower than 0.5% for resistance values below 10 GΩ. However, in the case of ultra-high resistance values, the measured value is highly dependent on the temperature. Figure 5 shows the resistance value measured for the 100 GΩ calibrated resistance in the decade box with different temperature and $V_{i}$ values. The circuit is stable for temperatures below $30{}^{\circ }C$ for all analysed $V_{i}$ values. However, for temperatures above $30{}^{\circ }C$, an increasing difference with respect to the real resistance value arises. These results make it necessary to introduce a correction factor in the case of high resistance and temperature, especially in the case of $V_{i}=5$ V. This correction factor is corrected in the digital part by the microcontroller since the variation is systematic.

## 6. Resistance Measurement in Timber Materials and Its Conversion to Internal Moisture Contents

Internal moisture content of certain materials is very important; a high moisture content might cause undesired biochemical and bacterial activity, modifying material characteristics. In the case of construction materials, moisture content (MC) also provides quality indication: a high moisture content in timber means poor quality since it might cause deformations and fissures. A good moisture measurement allows for optimizing selection, drying, storage, classification and distribution processes.

A common method to estimate moisture content in timber is using resistive methods [28]. It is well known that a direct relationship exists between the material resistance and the moisture content by means of conversion curves depending on the temperature and wood species [29,30]. Thus, an accurate equivalent resistance method is essential to obtain an equivalent moisture content estimation. However, there exist different factors in the measurements that makes moisture content estimation a complex process [31]: wood species, electronic circuit characteristics, temperature, measurement time, etc. In the proposed circuit design, we can easily adjust the system to user needs in the digital section by microcontroller reprogramming to include new material conversion curves, systematic corrections due to temperature, etc.

A European study about commercial moisture content devices for timber done in 2000 [32] stated that "most of the resistance meters showed a systematic deviation from the actual moisture content because of incorrect MC-resistance curves". This report showed that 90% of moisture content measurement devices do not meet the requirements of European Conformity (CE) marking in timber materials since accuracy in the value is above 2%. Additionally, these devices must be annually calibrated, according to European Norm EN14081–1:2006+A1:2011. In the case of resistive method devices, it must be done by evaluating the measurement with a resistance decade box jointly with the adequate conversion curve for each type of wood species.

In order to evaluate the validity of the developed circuit for moisture content estimation in timber materials, 20 timber pieces are subject to the same ambient conditions prior to and during the experiment. Different species were analysed: four pieces of oregon pine (*Pseudotsuga menziesii*, also known as Douglas fir), six pieces of cedar (*Cedrus atlantica*), six pieces of ash (*Fraxinus excelsior*) and four pieces of chestnut tree (*Castanea sativa*). Each timber piece was 90 mm× 35 mm × 45 mm, with two metallic stainless nails 20 mm apart as probes, inserted 10 mm. The probe distance to the borders was the same: 35 mm and 22 mm in length and width, respectively. Probes were placed in parallel to the grain. All timber pieces had fixed nails and the connection to the instrument was done using a detachable clamp.

To evaluate the drying process in timber over time, all pieces were submerged into water to guarantee a high moisture content, and, after that, they were placed under external ambient conditions without direct sun for 11 days, with an observation of the resistance value every 24 h. Each observation was the result of averaging 50 measured values for one minute. Thus, all timber pieces were measured after 20 min, guaranteeing the same ambient conditions for all of them. As different $V_{i}$ voltages were tested (5 V and 10 V), the same timber pieces with the same probe positions were used, completing the measurement in 40 min.

**Figure 6:**
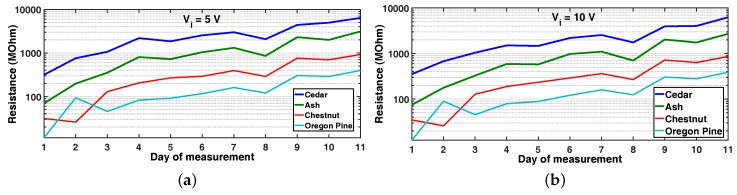
Resistance value evolution during an 11-day drying process in ambient conditions for Oregon pine, cedar, ash and chestnut tree timber for $V_{i}=5$ V (**a**) and 10 V (**b**).

Figure 6 shows the resistance value evolution for $V_{i}=5$ V and 10 V with temperature and relative ambient humidity range between $\left[26,\;29\right]{}^{\circ }C$ and [42, 74]%, respectively. As expected, since the timber pieces got dryer over time, the resistance values increased, and, in the case of day 8, which was a rainy day where ambient humidity increased (not direct rain on to the timber), a common variation was observed for all species. This shows that a correspondence between moisture content and resistance exists. It is important to note that the resistance value evolution is different according to the wood species, which confirms that a direct relationship between resistance and MC exists. Pine and chestnut show smaller resistance variation range compared with cedar and ash.

**Figure 7:**
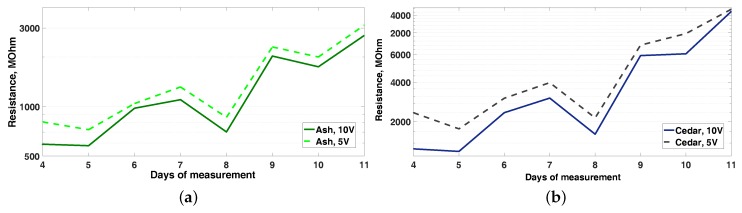
Comparison of measured resistance values for $V_{i}=5$ V and 10 V in ash tree (**a**) and cedar (**b**) wood types.

Concerning $V_{i}$, different values are used depending on the authors [33,34]. This analysis shows that the resistance evolution trend is the same for $V_{i}=$ 5 V and 10 V. However, some differences exist in resistance measured values for ash and cedar. Figure 7 shows the resistance values obtained in the case of ash between days 4 and 11. Resistance values are systematically higher in the case of $V_{i}=5$ V. These results lead to the need for developing specific MC-resistance analysis, not only dependent on the type of wood, but also on $V_{i}$.

## 7. Discussion

Through the different tests shown in this work, we have demonstrated that the proposed circuit is able to accurately obtain the equivalent resistance in materials. We obtained a maximum extended uncertainty of ±1 GΩ and a relative error lower than 1% (even in the case of large values such as 100 GΩ for temperatures below $30{}^{\circ }C$. Since accuracy is guaranteed, these results allow the application of the proposed circuit in material resistance measurement. The digital circuit section allows for including magnitude conversion curves, providing material characteristics as moisture content or any other physical or biochemical properties, and compensating for other systematic variations such as those due to temperature. Analysing the timber results, the circuit is able to follow the drying process of timber, which clearly reflects the capacity to correlate the resistance value with the moisture content.

To evaluate the circuit behaviour, different $V_{i}$ values are used, indicating that $V_{i}=5$ V can provide adequate results in all resistance ranges and temperatures as long as systematic variations are compensated. Then, we can assume that the circuit can be used for material resistance measurement, and, with proper addition of magnitude conversion procedures, multiple material properties can be estimated.

Concerning the thermal characterization of the circuit, we analysed the behaviour in the range $\left[10..50\right]{}^{\circ }C$. Results show that the accuracy is below 0.4% in the range [1 MΩ...10 GΩ]. In the case of resistance values above 10 GΩ, we tested 100 GΩ, showing that, above $35{\;}^{\circ }C$, the error can grow to up to 10%. However, this error is systematic and can be digitally compensated by the microprocessor since a temperature sensor is included in the circuit.

The results obtained in the drying process of timber materials over 11 days showed a direct relationship between the resistance and the moisture content. In addition, a clear difference among different timber materials was observed, which reflects the need for specific moisture conversion curves for each wood type or any other material, considering temperature and $V_{i}$ value for proper conversion.

In summary, the proposed circuit is able to accurately measure electrical resistance by means of a carefully designed and characterized analog circuit, and a digital circuit where the resistance value can be converted into any other value associated with a material property. As the proposed instrument is intended for multiple applications, specific conversion from the resistance value must be applied for material property magnitude by programming a conversion formula, typically associated with some linear or logarithmic relationship. This conversion procedure can be programmed in the digital section so that easy adaptation to different physicochemical property can be done. Thus, now that resistance value is accurately mesured, it is necessary to develop procedures to accurately convert the resistance value into any specific magnitude. It thus becomes possible to have a handheld device or wireless acquisition node due to its size and low power.

## 8. Conclusions

This work proposes a novel measurement system for wide range resistance measurement including ultra high resistance values up to 100 GΩ, showing accurate results for $R_{x}<10\;G\Omega$ (Table 3) and systematic deviations for higher values, which can be digitally compensated. Details on the circuit design are provided, analysing possible error sources when measuring a resistance $R_{x}$, and making experimental tests to show the validity of the circuit for long-term measurement, temperature variations and different timber materials. Furthermore, a novel circuit has been completed with additional circuitry for battery power supply, external communications to serve as a sensor node or a handheld device, and digital processing implementing magnitude conversion curves, averaging, and other systematic corrections as temperature. Thus, due to its flexibility in configuration, the proposed circuit can be applied to multiple materials where the internal resistance value is of interest. The circuit can be used standalone or as a part of a test bench by using the PC interface. This fact paves the way for different applications to detect the actual moisture content of processed wood, and building monitoring where timber materials are used. In addition, they often need verification of its moisture content due to possible damages in roofs or walls, calibration and verification of other instruments, material characterisation for temperature, humidity, chemicals, etc.

The dependence of timber resistance values for different $V_{i}$ (Figure 6) is also remarkable, which is not the case for a pattern resistance box. This means that $V_{i}$ affects the material properties, and it must be considered when converting a resistance value into moisture content or any other physicochemical magnitude. Thus, a more detailed study on this issue is required to confirm this effect.

## Figures and Tables

**Table 1:** Technical data of components used in the input amplifier for the theoretical characterization and experimental verification.

$R_{f}$	Operational Amplifier	MUX
Tolerance≤1%	$I_{b}\leq 30\;fA$	$R_{on}=5\;\Omega \ll R_{f}$
$TC\left(R_{f}\right)\leq 100\;\mathrm{ppm}{/}^{\circ }C$	$I_{os}\leq 30\;fA$	$I_{S}<10\;pA$
	$V_{os}\leq 2$ mV	$C_{D}<30\;pF$
	$TC\left(V_{os}\right)\leq 10\;\mu V{/}^{\circ }C$	$C_{S}<5\;pF$
	$\gamma_{n}\leq 85\;\mathrm{nV}/{\mathrm{Hz}}^{1/2}$	$C_{DS}<0.2\;pF$
	$\sigma_{n}\leq 0.1\;fA/{\mathrm{Hz}}^{1/2}$	

**Table 2:** Accuracy and extended uncertainty results showing the nominal value of the measured resistance ($R_{nom}$), the calibrated resistance value ($R_{cal}$), its tolerance, the obtained value using the measurement circuit ($R_{m}$), the extended uncertainty $\sigma_{ext}$ and the relative error $\epsilon_{r}$ when comparing the results with the calibrated value $R_{cal}$.

$R_{\mathrm{nom}}$	$R_{\mathrm{cal}}$	Accuracy (%)	$R_{m}$	$\sigma_{\mathrm{ext}}$	$\epsilon_{r}\left(\%\right)$
1 MΩ	1.0000057 MΩ	±0.00002	1.0049 MΩ	±0.0004	0.5
10 MΩ	10.000028 MΩ	±0.0005	10.083 MΩ	±0.045	0.8
100 MΩ	100.00173 MΩ	±0.01	100.4211 MΩ	±0.0001	0.4
1 GΩ	1.000183 GΩ	±0.005	1.0042 GΩ	±0.0001	0.4
10 GΩ	10.016717 GΩ	±0.005	10.0436 GΩ	±0.0012	0.3
100 GΩ	99.89014 GΩ	±1	99.235 GΩ	±1.010	0.7

**Table 3:** Resistance values measured in a climate chamber with temperature variations in the range $\left[10,\;50\right]{}^{\circ }C$ for $R_{x}$ resistance values of [1 MΩ...10 GΩ] with power supply $V_{i}=5$ V.

	$R_{\mathrm{cal}}$	1.0000057 MΩ	10.000028 MΩ	100.00173 MΩ	1.000183 GΩ	10.016717 GΩ	
T (${}^{\circ }$C)							
10		0.9968	10.0119	99.7234	0.997230	9.9723	$R_{m}$
		0.0002	0.0053	0	0	0	$\sigma_{ext}$
		0.3	0.1	0.3	0.3	0.4	$\epsilon_{r}\;\left(\%\right)$
20		0.9971	10.0150	99.7234	0.997234	9.9723	$R_{m}$
		0.0006	0	0	0	0	$\sigma_{ext}$
		0.3	0.1	0.3	0.3	0.4	$\epsilon_{r}\;\left(\%\right)$
30		0.9981	10.0058	99.7234	0.997234	9.9723	$R_{m}$
		0	0.0181	0	0	0	$\sigma_{ext}$
		0.2	0.06	0.3	0.3	0.4	$\epsilon_{r}\;\left(\%\right)$
40		0.9981	10.0080	99.7234	0.997277	10.0150	$R_{m}$
		0	0.0159	0	0.004305	0	$\sigma_{ext}$
		0.2	0.08	0.3	0.3	0.02	$\epsilon_{r}\;\left(\%\right)$
50		0.9981	9.9779	99.7234	0.997277	10.0150	$R_{m}$
		0	0.0145	0	0.0004283	0	$\sigma_{ext}$
		0.2	0.2	0.3	0.3	0.2	$\epsilon_{r}\;\left(\%\right)$
